# Gastrin inhibits gastric cancer progression through activating the ERK-P65-miR23a/27a/24 axis

**DOI:** 10.1186/s13046-018-0782-7

**Published:** 2018-06-04

**Authors:** Li-Dong Zu, Xing-Chun Peng, Zhi Zeng, Jing-Long Wang, Li-Li Meng, Wei-Wei Shen, Chun-Ting Hu, Ye Yang, Guo-Hui Fu

**Affiliations:** 1 Pathology Center, Shanghai General Hospital/Faculty of Basic Medicine, Key Laboratory of Cell Differentiation and Apoptosis of Chinese Ministry of Education, Institutes of Medical Sciences, Shanghai Key Laboratory of Gastric Neoplasms, Shanghai Institute of Digestive Surgery, Ruijin Hospital, Shanghai Jiao Tong University School of Medicine, Shanghai, China; 2 Department of Pathology, Renmin Hospital of Wuhan University, Wuhan, China; 3 Department of Digestive Medicine, Ningbo No. 2 Hospital, Ningbo, 315010 China; 4 Pathology Center, Shanghai General Hospital/Faculty of Basic Medicine, Shanghai Jiao Tong University School of Medicine, No. 280, South Chong-Qing Road, Shanghai, 200025 People’s Republic of China

**Keywords:** Gastric cancer, Gastrin, ERK, P65, miR23a/27a/24 cluster

## Abstract

**Background:**

To test the hypothesis that activated extracellular signal-regulated kinase (ERK) regulates P65-miR23a/27a/24 axis in gastric cancer (GC) and the ERK-P65-miR23a/27a/24 axis plays an important role in the development of GC, and to evaluate the role of gastrin in GC progression and ERK-P65-miR23a/27a/24 axis.

**Methods:**

The component levels of the ERK-P65-miR23a/27a/24 axis in four fresh GC tissues, 101 paraffin-embedded GC tissues and four GC cell lines were determined by Western blotting, immunohistochemistry (IHC) or qRT-PCR. The effects of gastrin on GC were first evaluated by measuring gastrin serum levels in 30 healthy and 70 GC patients and performing a correlation analysis between gastrin levels and survival time in 27 GC patients after eight years of follow-up, then evaluated on GC cell lines, GC cell xenograft models, and patient-derived xenografts (PDX) mouse models. The roles of ERK-P65-miR23a/27a/24 axis in GC progression and in the effects of gastrin on GC were examined.

**Results:**

ERK- P65-miR23a/27a/24 axis was proved to be present in GC cells. The levels of components of ERK-P65-miR23a/27a/24 axis were decreased in GC tissue samples and PGC cells. The decreased levels of components of ERK-P65-miR23a/27a/24 axis were associated with poor prognosis of GC, and ERK-P65-miR23a/27a/24 axis played a suppressive role in GC progression. Low blood gastrin was correlated with poor prognosis of the GC patients and decreased expression of p-ERK and p-P65 in GC tissues. Gastrin inhibited proliferation of poorly-differentiated GC (PGC) cells through activating the ERK-P65-miR23a/27a/24 axis. Gastrin inhibited GC growth and enhanced the suppression of GC by cisplatin in mice or PGC cell culture models through activating the ERK-P65-miR23a/27a/24 axis or its components.

**Conclusions:**

ERK-P65-miR23a/27a/24 axis is down-regulated, leading to excess GC growth and poor prognosis of GC. Low gastrin promoted excess GC growth and contributed to the poor prognosis of the GC patients by down-regulating ERK-P65-miR23a/27a/24 axis. Gastrin inhibits gastric cancer growth through activating the ERK-P65-miR23a/27a/24 axis.

**Electronic supplementary material:**

The online version of this article (10.1186/s13046-018-0782-7) contains supplementary material, which is available to authorized users.

## Background

Gastric cancer (GC) is the leading cause of cancer-related mortality worldwide and remains a considerable health burden throughout the world. Surgery is the only curative treatment. For locally advanced disease, adjuvant or neoadjuvant therapy is usually implemented in combination with surgery. Outcomes in metastatic disease are poor, with median survival being around 1 year. Despite progress in deciphering its development, challenges with GC treatment remain. Many patients have inoperable disease at diagnosis or have recurrent disease after resection with curative intent [1–4].

Gastric cancer is histologically classified into diffuse and intestinal types, termed PGC (poorly-differentiated GC) and WGC (well-differentiated GC), respectively [1–4]. Chronic atrophic gastritis (CAG) and WGC are developed after over-time inflammatory and wound-healing responses triggered by chronic gastric injury of any etiology [5]. Molecular studies have demonstrated that progression of WGC and PGC may have different molecular pathologies, although the underlying mechanisms are not completely understood. To achieve good prognosis of GC therapy, elucidation of mechanism for GC pathogenesis is imperative.

The NF-κB P65 subunit (P65) is expressed in nearly all cell types [6], and is known to regulate the expression of many genes that are involved in a variety of cellular responses including inflammation, immunity, cell proliferation and apoptosis [7–10]. The transcriptional activity of P65 is enhanced by ERK signaling-mediated phosphorylation (p-P65), which also increases P65 protein stability [11–13]. P65 was once considered to be an oncogene in several types of solid tumors [14–16]. Extracellular signal-regulated kinase 1 and 2 (ERK1/2) are serine/threonine kinases and part of the Ras-Raf-MEK-ERK signal (mitogen-activated protein kinase (MAPK) signal pathway) transduction cascade, transmitting signals from cell surface receptors to regulate proliferation, differentiation, and survival programs. They also play a central role in the development of human cancer [17]. ERK signaling is activated in more than 30% of human cancers, most frequently via RAS (rat sarcoma virus) and BRAF (v-Raf murine sarcoma viral oncogene homolog B) mutations [18–20]. Inhibitors targeting ERK signaling can be used as cancer therapeutic agents [21–23]. More than 29 kinds of kinase inhibitors have been developed to treat various cancers, including the BRAF inhibitors vemurafenib and dabrafenib of ERK signaling and the MEK (Mitogen-activated protein kinase kinase) inhibitor trametinib [24–26].

In our previous study, we have shown that p-P65 binds to the promoter region of the miR23a/27a/24 cluster and potently up-regulates miR-23a, miR-27a, and miR-24 expression that is linked to differentiation of erythroid-directed hemopoietic stem cells (HSC) [27]. This cluster was the first downstream miRNA target implicated in regulating the development of myeloid versus lymphoid cells [28]. Recently, altered expression of the miR23a/27a/24 cluster was found to be associated with solid tumors [29], and P65 is phosphorylated by the MAPK pathway [11–13], suggesting a potential association between the terminal Ser/Thr kinase ERK and the P65-miR23a/27a/24 cluster.

Gastrin, a peptide hormone, is synthesized in the G cells of the antrum; however, gastrin expression also is found in many gastric adenocarcinomas of the stomach corpus. Gastrin’s actions are mediated through the G-protein-coupled receptor cholecystokinin-B (CCK-B) on parietal and enterochromaffin cells of the gastric body. In a previous study, we have shown that gastrin inhibits PGC growth in vitro and in vivo [30]. Several studies based on clinical observation or animal models of hypergastrinemia have shown that gastrin promoted tumor growth, and there is no precise assessment of how gastrin contributes to GC progression in humans [31–34].Whether and how gastrin affects GC cells remains controversial [35–37].

In this study, we hypothesized that activated ERK regulated P65-miR23a/27a/24 axis in GC and the ERK-P65-miR23a/27a/24 axis played an important role in GC progression. We tested this hypothesis and evaluated the role of gastrin in GC progression and modulating ERK-P65-miR23a/27a/24 axis. Our data indicated that gastrin inhibited GC progression and activated ERK-P65-miR23a/27a/24 axis which functioned as a GC suppressor. Gastrin and the ERK-P65-miR23a/27a/24 axis could be a potential drug target for PGC treatment.

## Methods

### Human tissue samples

Human gastric cancer tissue samples and para-tumor tissue samples were obtained from the Department of Digestive Surgery, Ruijin Hospital, School of Medicine, Shanghai Jiao Tong University. These samples were immediately frozen in tubes and stored in liquid nitrogen after surgical resection on the PGC patients diagnosed by clinical pathologists. Four GC samples with upfront neoadjuvant chemotherapy were collected from Pathology Center, Shanghai General Hospital/Faculty of Basic Medicine, Shanghai Jiao Tong University School of Medicine. Peripheral blood samples were collected from GC patients treated at Ruijin Hospital and Lishui Hospital, Zhejiang province during 2009–2010. These patients had not received chemotherapy or radiotherapy before surgery. Venous blood (3 ml) was collected from fasting patients into endotoxin- and pyrogen-free test tubes. Serum samples were further collected to Eppendorf tubes and stored at − 80 °C until analysis. Patient survival was followed up through visiting until October 2016.Written informed consent was obtained from each patient. This study was approved by the Ethics Committee of Shanghai Jiao Tong University School of Medicine.

### Xenograft GC nude mice model

Female athymic BALB/c nude mice (6–8 weeks old) were purchased from Shanghai Experimental Animal Center, Chinese Academy of Science. The nude mice were subcutaneously injected with 5×10^6^ SGC7901 cells suspended in PBS (phosphate buffer saline) and grew until the tumors reached ~ 200 mm^3^. These mice were randomly divided into five groups and treated with LPS (lipopolysaccharide, 1 mg/kg/3d, enterocoelia), BA (Betulinic acid, 20 mg/kg/3d, intragastric), miRNA mimics (10 μg/week/mouse, tumor), gastrin (2 mg/kg, twice/day, subcutaneous), and PBS once daily (*n* = 6 for each group) for 14 days. These mice were also randomly divided into four groups and treated with ERK inhibitor PD98059 (10 mg/kg/day, once per 3 days, Selleck), P65 inhibitor PN (Parthenolide, 4 mg/kg/day, once per day, Selleck), miR23a/27a/24 inhibitors (10 μg/week/mouse, GenePharma), and the vehicle control for 14 days. All mice were sacrificed after anesthesia and tumor size and weight were measured. Tumor volume was calculated, V = length×width^2^/2 mm^3^. The experiments were approved by the animal research committee in Shanghai Jiao Tong University.

### GC patient-derived xenograft (PDX) mice model

Patient-derived tumor tissues were collected in culture medium and kept on ice for engraftment within 24 h of resection. Necrotic and supporting tissues were carefully removed using a surgical blade. A piece of tissue approximately 20–30 mg in weight was cut and implanted subcutaneously into the flank region of athymic nude female mice using a trocar. GC PDX mice were randomly divided into two groups and treated by subcutaneous injection with gastrin (2 mg/kg) or 100 μl PBS twice daily. Gastrin (pGlu-GPWLEEEEEAWGWMDF-NH2, designated as Gastrin) was obtained from China Peptides (Shanghai, China). The experiments were approved by the animal research committee in Shanghai Jiao Tong University.

### Tissue microarray assay

GC tissue microarray (TMA) assay was performed in our lab by following the published procedure [38]. To prepare TMA, a total of 101 GC specimens (35 WGC and 66 PGC tissue samples and their corresponding normal para-tumor tissues) were included and duplicate 1.0 mm cores were collected by punching each paraffin tumor or para-tumor tissue sample block in the training cohort or the validation cohorts. As a control, the normal gastric epithelium tissues were inserted in the four corners and in the center of each slide. Upon Hematoxylin and Eosin staining, TMA was examined by two senior pathologists for diagnosis of WGCs and PGCs. Tumor histological classification was assessed according to the World Health Organization criteria. TNM (tumor, node, metastasis) staging was classified according to the manual of the International Union Against Cancer/American Joint Committee on Cancer (2010).

### Cell culture

Human GC lines (SGC7901, AGS, MKN45, and MKN28) were purchased from the Cell Bank of the Shanghai Institute for Biological Science (Shanghai, China) and cultured in RPMI-1640 (Hyclone, Thermo Fisher, USA) medium supplemented with 10% fetal bovine serum (FBS) (Hyclone) and 1% penicillin/streptomycin (Invitrogen, Carlsbad, CA, USA) at 37 °C in a humidified atmosphere with 5% CO_2_. All cells used in the experiments were in the exponential growth phase.

### Determination of relative miRNA levels using quantitative real time PCR (qRT-PCR)

Total RNA was extracted from cells or GC tissue samples after homogenization using Trizol reagent (Invitrogen) according to the manufacturer’s instructions, and were reverse transcribed (TaKaRa) into cDNA with specific RT primers. The relative miRNA levels were analyzed by qRT-PCR using a One Step SYBR PrimeScript™ RT-PCR Kit II (TaKaRa) and an ABI 7500 fast fluorescence temperature cycler. U6 was used for normalization. The relative levels of each microRNA were calculated using the 2^−ΔΔCt^ method after normalization. All experiments were repeated three times. The primer sequences were:

**Table:** 

U6 RT	CTCAACTGGTGTCGTGGAGTCGGCAATTCAGTTGAGAAAATATG
miR-23a RT	CTCAACTGGTGTCGTGGAGTCGGCAATTCAGTTGAGGGAAATCC
miR-27a RT	CTCAACTGGTGTCGTGGAGTCGGCAATTCAGTTGAGGCGGAACT
miR-24 RT	CTCAACTGGTGTCGTGGAGTCGGCAATTCAGTTGAGCTGTTCCT
U6 forward	ACACTCCAGCTGGGCGCAAATTCGTGAAGC
miR-23a forward	ACACTCCAGCTGGGATCACATTGCCAGGG
miR-27a forward	ACACTCCAGCTGGGTTCACAGTGGCTAAG
miR-24 forward	ACACTCCAGCTGGGTGGCTCAGTTCAGCAG
universal reverse	CTCAACTGGTGTCGTGGAGTCGG

### Western blotting

Whole cell lysates were prepared in RIPA buffer (Thermo Scientific) with phenylmethylsulfonyl fluoride and protease inhibitors and centrifuged. Supernatants were aliquoted, mixed with loading buffer, resolved by 10% sodium dodecyl sulfate–polyacrylamide gel electrophoresis and then transferred onto polyvinylidenedifluoride membranes (Millipore, Billerica, MA). After blocked with 5% skim milk in TBST (Tris-buffered Saline with Tween 20) at room temperature for 1 h, the membranes were incubated with different primary antibodies, including anti-ERK, anti-p-ERK, anti-P65, anti-p-P65, anti-cyclin D1 (1:1000,Cell Signaling Technology, Danvers, MA, USA), and anti-GAPDH (1:5000, Yeason, China) in 5% milk/TBST buffer at 4 °C overnight, and then probed with horseradish peroxidase-conjugated anti-mouse or anti-rabbit IgG (1:5000, Jackson Immunoresearch Laboratories, West Grove, PA, USA) for 1 h. After washing with TBST, the membrane was developed with enhanced chemiluminescent plus substrate (Merck Millipore, Billerica, MA, USA) and the signal was recorded by Fluorchem E System (Protein Simple, Santa Clara, CA, USA).

### In vitro cell proliferation assay

The cell proliferation assay was performed using a CCK-8 (Cell Counting Kit-8) kit (Dojindo, Japan). GC cells (MKN28, SGC7901, AGS, and MKN45) were seeded at a density of 2 × 10^3^ cells per well into 96-well plates with each well containing 100 μl medium. After culture for 24 h, the GC cells were treated with lipopolysaccharides (LPS), PD98059, betulinic acid (BA), and parthenolide (PN) (Sigma Chemical Co., St. Louis, MO, USA), and incubated for desired duration. The OD value of each well was measured at 450 nm.

The cell proliferation was also assayed by cell counting with trypan blue. SGC7901 cells were seeded into 12-well plates. After desired treatment, the cells were washed twice with PBS and treated with trypsin at 37 °C for 1 min. RPMI 1640 containing 10% FBS was added, mixed, and centrifuged. The cells were resuspended with RPMI 1640 containing 0.4% trypan blue and counted in a blood cell counting chamber.

### ELISA (enzyme linked immunosorbent assay)

Gastrin levels were determined using a Human Gastrin ELISA kit (Shanghai yuan Mu Biotechnology Co., Ltd., Shanghai, China) following the manufacturer’s procedure. The enzyme-catalyzed reaction was stopped with 2 M H_2_SO_4_ and read using a microplate reader (Thermo Fisher Scientific, Waltham, MA, USA) at 450 nm.

### Immunohistochemistry

Tumor specimens were fixed in 10% formalin overnight and embedded in paraffin. To observe ERK1/2 and P65 expression in gastric carcinoma, deparaffinized slides were treated with 3% H_2_O_2_ and subjected to antigen retrieval using 0.01 M citric buffer solution (pH 6.0). After overnight incubation with the indicated primary antibody at 4 °C, the slides were incubated for 15 min at room temperature with horseradish peroxidase-labeled polymer conjugated to a secondary antibody (Max Vision™ Kit) and incubated with diaminobenizidine (DAB) for 2 min. The slides were then counterstained with Hematoxylin and Eosin. Appropriate positive and negative controls were tested in parallel. All slides were evaluated by three independent observers who were unaware of the disease outcome. For ERK1/2 and P65, less than 10% of expression was considered to be “loss” (−), and more than 10% of expression was designated (+).

### In situ hybridization

Frozen tissue sections were first digested with 5 mg/ml proteinase K at room temperature for 5 min and then loaded onto a Ventana Discovery Ultra unit. The tissue slides were incubated with double digoxidenin (DIG)- labeled mercury LNA (locked nucleic acid) miR-23a-3p probe, miR-27a-3p probe, miR-24-3p probe, or U6 snRNA probe (Exiqon) at 45 °C for 2 h. The digoxigenin label was then detected with a polyclonal anti-DIG antibody conjugated with alkaline phosphatase using NBT-BCIP (nitroblue tetrazolium/5-Bromo-4-Chloro-3-Indolyl Phosphate) as the substrate. The signal intensities for miR-23a, miR-27a, miR-24, and U6 were quantified using the Image-Pro Plus software package (Media Cybernetics) as reported previously [39].

### Reporter gene assay

Human Cyclin D1 3′-untranslated region (UTR) containing putative miR-23a, miR-24 and miR-27a binding sites was amplified by PCR from human genomic DNA and digested with restricted enzyme Not I and Xba I, and then cloned into pRL-TK (Promega). 293 T cells were co-transfected with this plasmid andmiR-23a, miR-24, miR-27a mimics, or the siRNA control and incubated for 48 h. Cells were collected for the luciferase activity assay using a dual-luciferase reporter assay system (Promega) according to the manufacturer’s instructions. The pGL-3 plasmid (firefly) was also cotransfected as an endogenous control for normalization.

### Statistical analysis

Statistical analysis was performed using SPSS 19.0 software system (SPSS Inc., Chicago, IL, USA). Student’s t-test was performed for continuous variables and a chi-square test was used to analyze the differences of categorical variables. Univariate survival analysis was carried out by the Kaplan-Meier method and evaluated using the log-rank test. *P* < 0.05 was considered significant.

## Results

### ERK regulated P65 activity and miR23a/27a/24 levels in GC cells

**Fig. 1:**
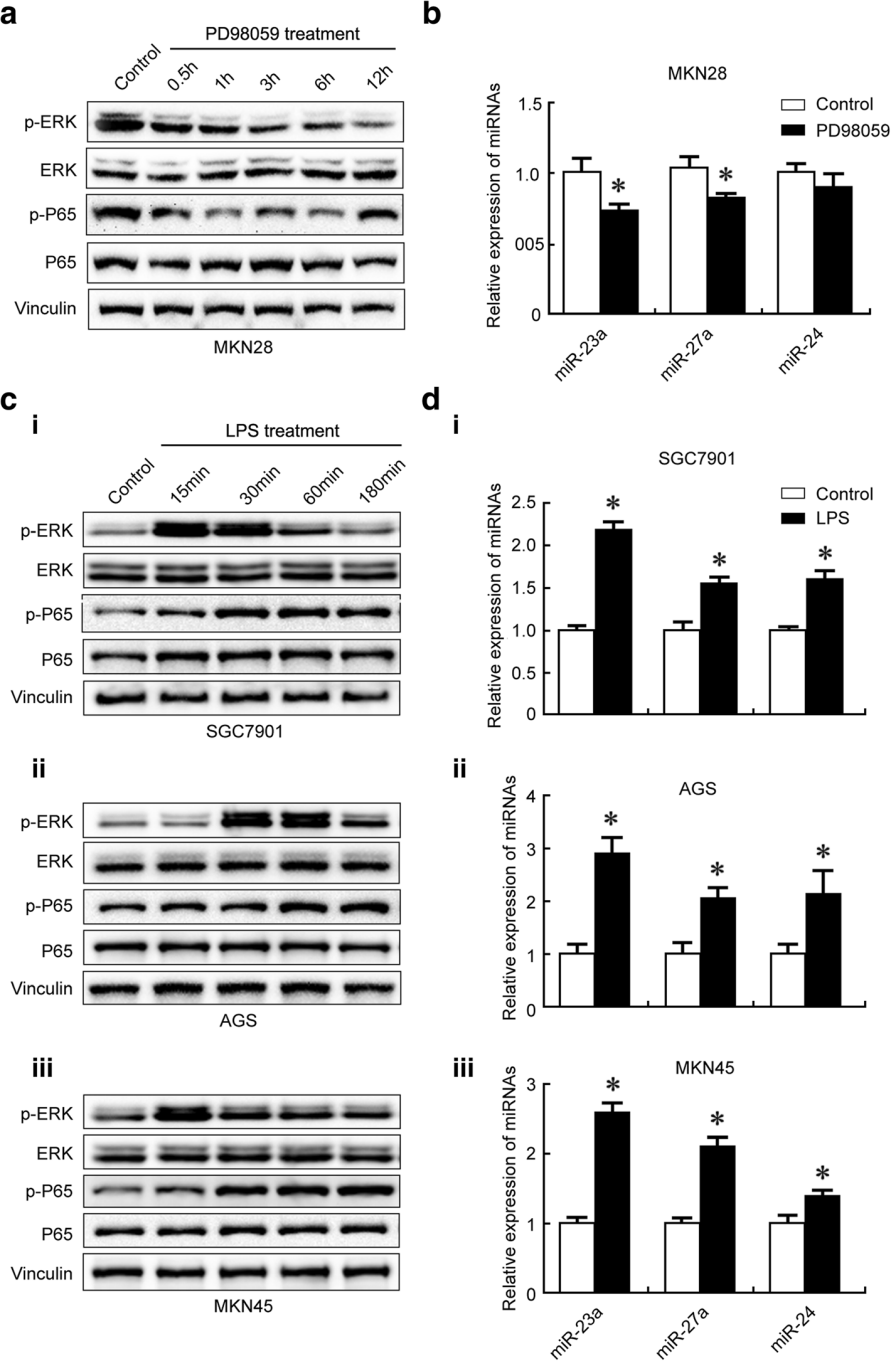
ERK regulated P65 activity and miR23a/27a/24 levels in GC cells. The p-P65 level (**a**) and miR-23a, miR-27a and miR-24 levels (**b**) were decreased in MKN28 cells after PD98059 treatment. The p-P65 level (**c**) and miR-23a, miR-27a and miR-24 levels (**d**) were increased in PGC cells (SGC7901, AGS and MKN45) after LPS treatment. Protein levels were determined using Western blotting (**a** and **c**) with Vinculin as a loading control. MicroRNA levels were determined using qRT-PCR with U6 as a normalization control (**b** and **d**). The fold change was calculated using the 2^−ΔΔCt^ method. *, compared with the control, *p*<0.05. All experiments were repeated three times

To examine whether ERK regulates P65-miR23a/27a/24 in GC, we treated GC cells with the ERK inhibitor PD98059 [40] or ERK activator LPS [41] and determined the levels of p-ERK, p-P65, miR-23a, miR-27a, and miR-24. The results showed that p-ERK, p-P65 miR-23a, miR-27a and miR-24 levels were decreased in MKN28 cells (WGC cells) after PD98059 treatment (Fig. 1a-b). They were increased in PGC cells (SGC7901, AGS, and MKN45) after LPS treatment (Fig. 1c-d). The results suggested the presence of ERK-P65-miR23a/27a/24 axis in GC and ERK regulated P65 activity and miR23a/27a/24 levels in GC cells.

### The levels of components of ERK-P65-miR23a/27a/24 axis were decreased in GC tissue samples and PGC cells and associated with poor prognosis of GC

**Fig. 2:**
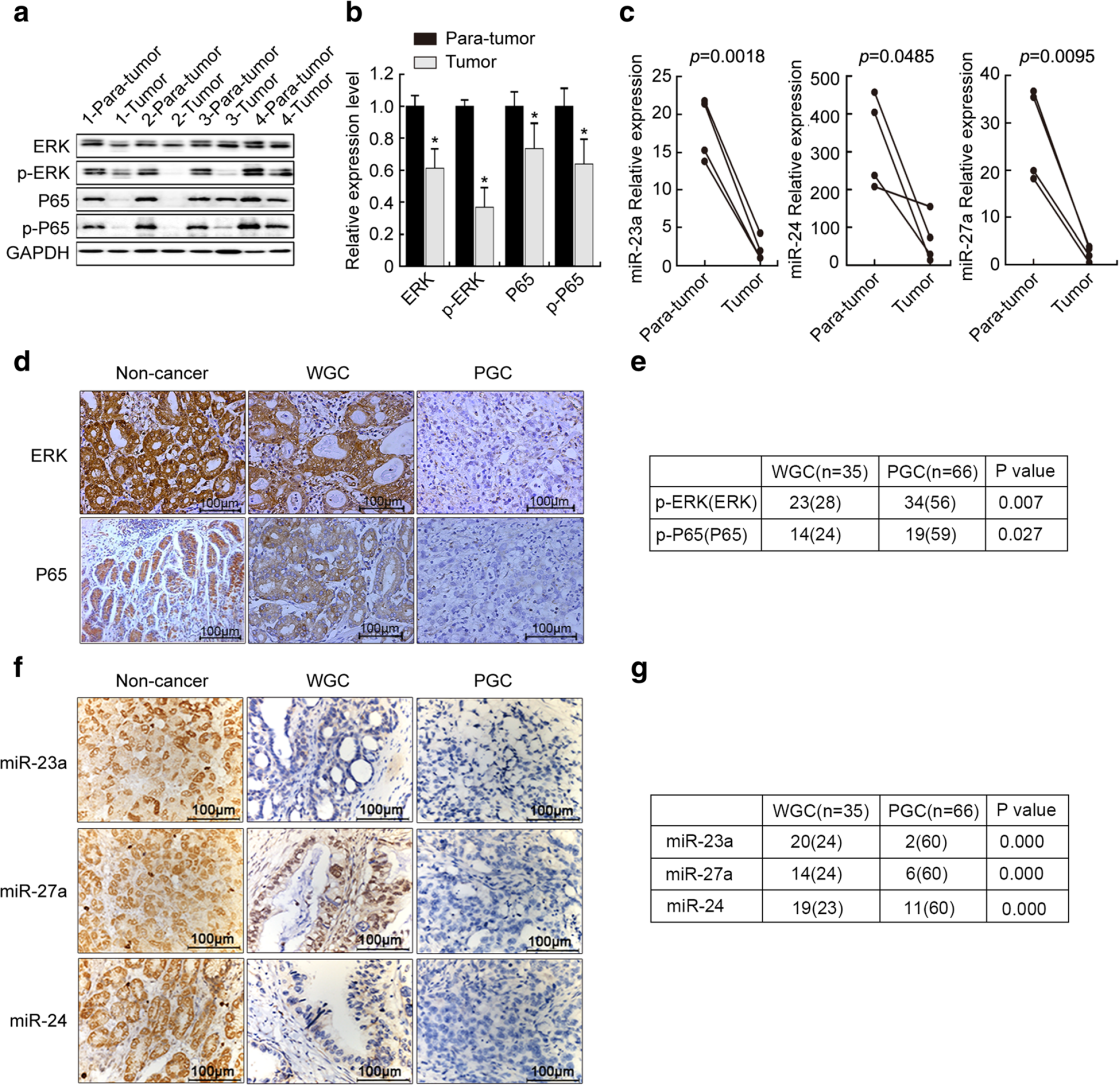
The levels of the components of ERK-P65-miR23a/27a/24 axis were decreased in GC tissue samples. **a** The relative ERK, P65, p-ERK, and p-P65 protein levels in GC tissue samples, which were determined by Western blotting and quantification of density of the bands in using Image J software (**b**). Each band was normalized to GAPDH in the same tissue. The levels of ERK, P65, p-ERK, and p-P65 in tumor tissues were normalized to those para-tumor tissues. *, compared to the para-tumor tissues, *p*<0.05. **c** The miR-23a, miR-27a and miR-24 levels in GC tissue samples, which were determined using qRT-PCR and normalized to U6. Fold-change was calculated using the 2^−ΔΔCt^ method. The *p* values were analyzed using GraphPad 5.0 software. *, comparison between the tumor and para-tumor tissues, *p*<0.05. **d** The expression of ERK and P65 in human gastric tissue samples. ERK and P65 expression were analyzed using immunohistochemistry on a microarray of human normal, WGC, and PGC tissues using anti-ERK and anti-P65 antibodies. Representative microscopic images (Scale bar, 100 μm) of staining were shown. **e** Relative frequency of positive ERK and P65 expression in WGC and PGC tissues. **f** The relative miR-23a, miR-27a and miR-24levels in human gastric tissue samples. The microRNA levels were analyzed using in situ hybridization on a microarray of human normal, WGC, and PGC tissues using pertained probes. Representative microscopic images (Scale bar, 100 μm) of staining. **g** Relative frequency of positivemiR-23a, miR-27a and miR-24 expression in WGC and PGC tissues

**Fig. 3:**
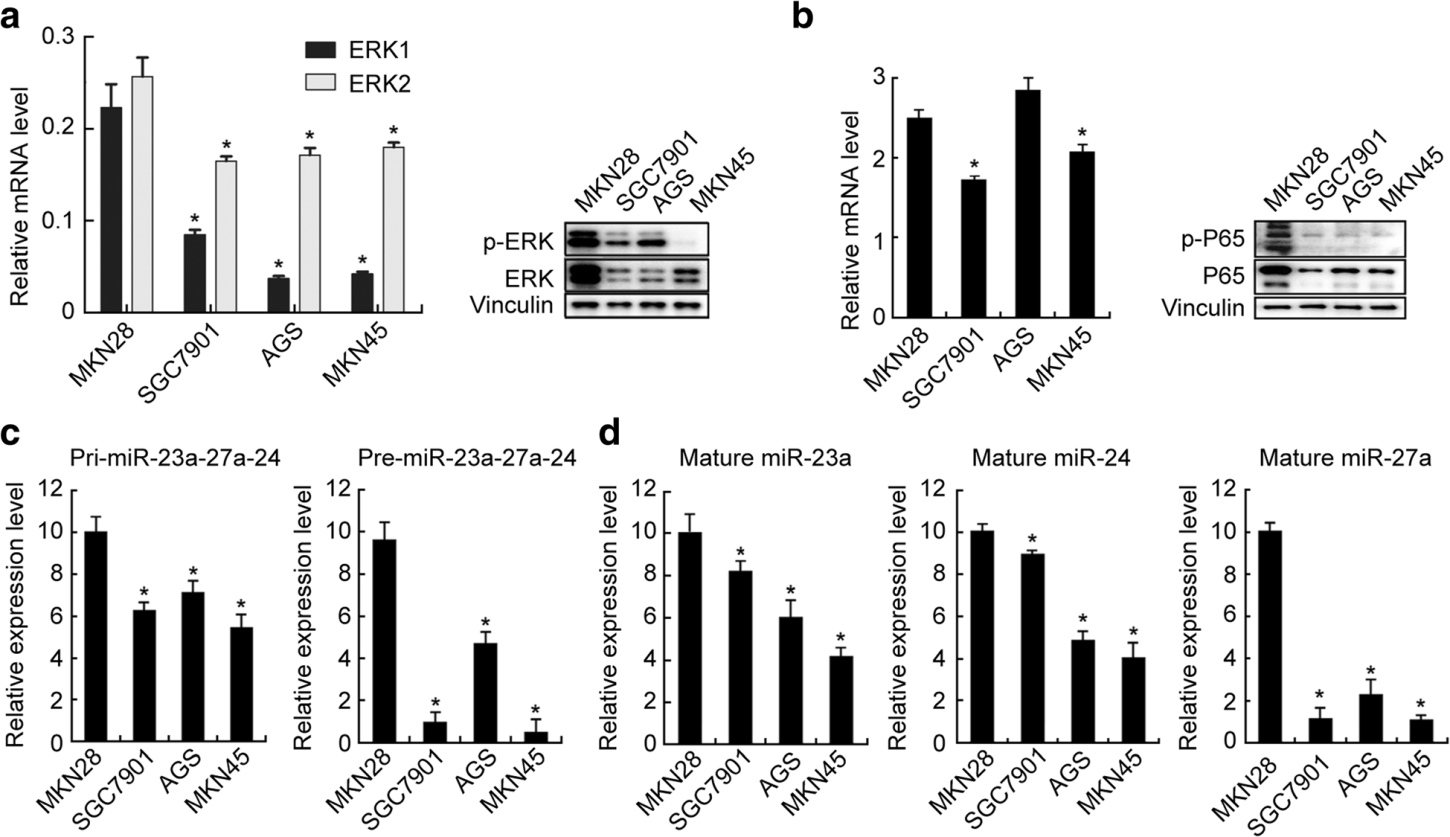
The levels of the components of ERK-P65-miR23a/27a/24 axis were decreased in PGC and WGC cells. **a** The ERK1/2 and **b** P65mRNA and protein levels in PGC and WGC cells, which were determined using qRT-PCR and Western blotting, respectively. The mRNA levels were normalized to GAPDH. The experiments were repeated three times. *, compared with the MKN28 group, *p*<0.05. **c**, **d** The pre-miR23a/27a/24, miR-23a, miR-27a, and miR-24 levels in PGC and WGC cells, which were determined using qRT-PCR and normalized to U6. Fold-change was calculated using the 2^−ΔΔCt^ method. *, compared with the MKN28 group, *p*<0.05. All experiments were repeated three times

To examine the potential association of the ERK-P65-miR23a/27a/24 axis with GC progression, we determined the levels of the components of ERK-P65-miR23a/27a/24 axis in the GC tissue samples and PGC and WGC cells using Western blotting, qRT-PCR, IHC and in situ hybridization microarrays. The results showed that ERK, p-ERK, P65, p-P65, miR-23a, miR-27a, and miR-24 levels were significantly decreased in fresh tumor tissues from four PGC patients, compared with those of the para-GC tissues (Fig. 2a-c). ERK and P65 were highly expressed in non-cancer gastric epithelium and WGC tissues, but were significantly decreased in PGC tissues (Fig. 2d). ERK and P65 were more frequently expressed in WGC than PGCs (Fig. 2e), so did with miRNAs miR-23a, miR-27a, and miR-24 in non-cancer and WGC tissues than PGC tissues (Fig. 2f and g). Consistently, both ERK mRNA and protein and p-ERK protein levels were lower in PGC cells (SGC7001, AGS, and MKN45) than those in WGC cells (MKN28) (Fig. 3a). So did with P65and p-P65 protein levels, whereas P65 mRNA levels were slightly changed (Fig. 3b). pri-miR23a/27a/24, pre-miR23a/27a/24, miR-23a, miR-27a, and miR-24 levels were significantly lower in PGC cells than WGC cells (Fig. 3c, d). These results indicated that the levels of components of ERK-P65-miR23a/27a/24 axis were decreased in GC tissue samples and PGC cells, compared with the para-tumor tissues and WGC, respectively, suggesting that the ERK-P65-miR23a/27a/24 axis was associated with GC progression.

We further performed an association analysis between the levels of components of ERK-P65-miR23a/27a/24 axis and clinicopathological characteristics of GC patients. The results showed that ERK and P65 expression were more frequently found in tumors not more than 4 cm in diameter, WGC tissues, and intestinal type GC tissues (Additional file 1: Tables S1 and Additional file 2: Table S2). These data suggested that high levels of the components of the ERK-P65-miR23a/27a/24 axis were associated with good prognosis of GC.

### Suppression of GC growth by ERK-P65-miR23a/27a/24 axis

**Fig. 4:**
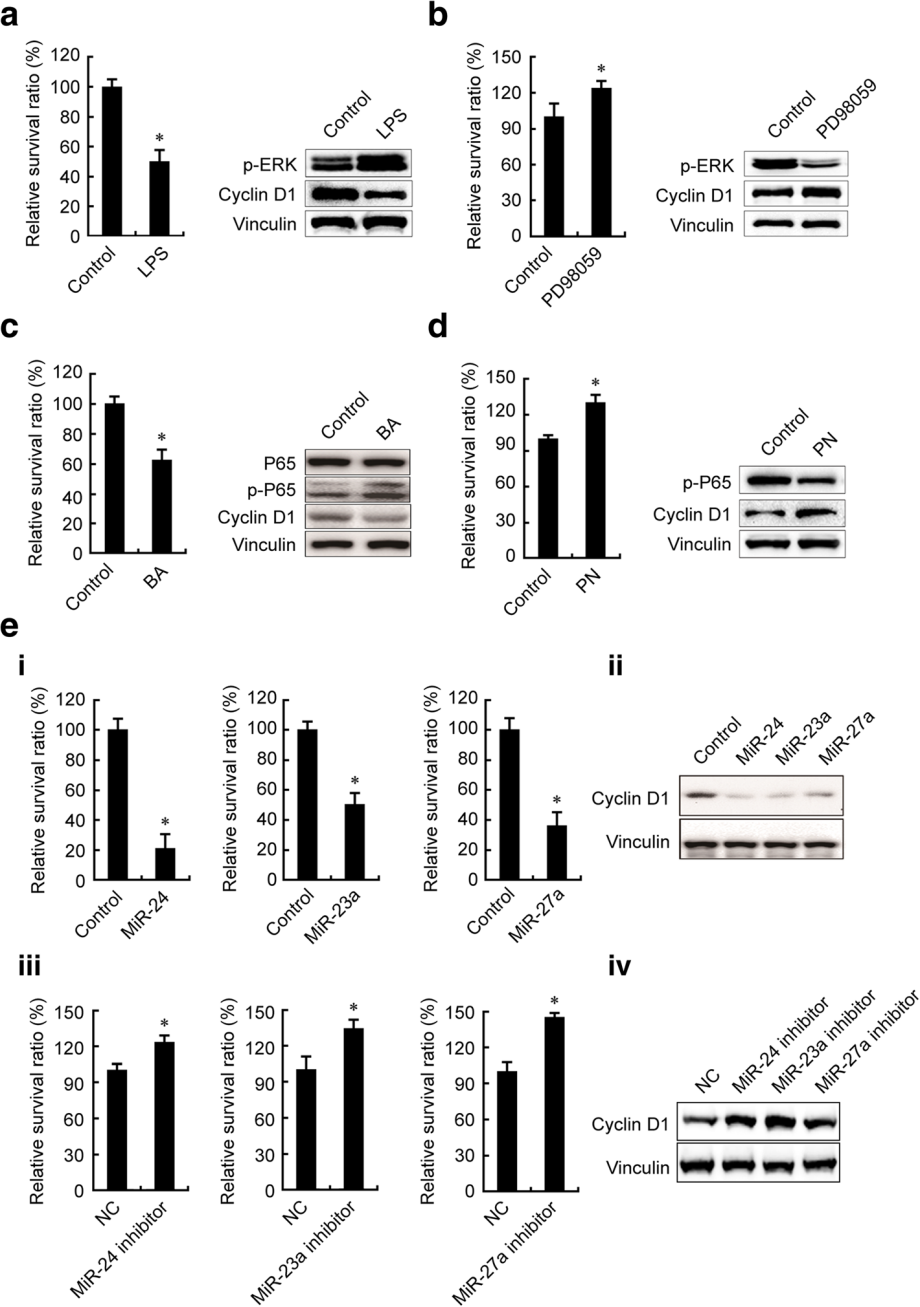
Modulators of ERK-P65-miR23a/27a/24 axis regulated GC proliferation. The p-ERK and Cyclin D1 levels and proliferation of SGC7901 (**a**) and MKN28 (**b**) cells were modulated by LPS (**a**) and PD98059 (**b**). The p-P65 and Cyclin D1 levels and proliferation of SGC7901 (**c**) and MKN28 (**d**) cells were modulated by BA (**c**) and PN (**d**). The Cyclin D1 levels and proliferation of SGC7901 (**e**-I, II) and those of MKN28 (E-III, IV) were modulated by miR23a/27a/24 mimics (**e**-I, II) and miR23a/27a/24 inhibitors (**e**-III, IV). SGC7901 and MKN28 cells were treated with the modulators as indicated for 48 h. The p-ERK, Cyclin D1, and p-P65 protein levels were determined using Western blotting. Proliferation of SGC7901 and MKN28 cells were determined by living cell counting. *, compared with the control group, *p*<0.05. All experiments were repeated three times

To examine whether the components of the ERK-P65-miR23a/27a/24 axis regulated GC growth, we first treated PGC cells (SGC7901) cells with LPS or the P65 activator BA [42] and WGC cells (MKN28) with the ERK inhibitor PD98059 or the P65 inhibitor PN [43] and determined the changes in cell proliferation and Cyclin D1 expression. The results showed that LPS and BA up-regulated p-ERK and p-P65 levels as expected, and proliferation of SGC7901 cells and Cyclin D1 expression were inhibited by both LPS and BA (Fig. 4a and c). PD98059 or PN decreased p-ERK and p-P65 levels as expected and enhanced cell proliferation and Cyclin D1 expression (Fig. 4b and d). Further, in order to exclude the off-target effect of PD98059 on p38 and JNK pathway, we examined the effect of ERK specific inhibitor LY3214996 on the proliferation of MKN45 cells. The results showed that LY3214996 promoted the proliferation of MKN45 GC cells (Additional file 3: Figure S1).

We transfected SGC7901 cells with miR-23a, miR-27a, and miR-24 mimics and found that these mimics inhibited cell proliferation and Cyclin D1 expression of SGC7901 cells (Fig. 4e, I and II). We transfected MKN28 cells with miR-23a, miR-27a, and miR-24 inhibitors and found that these inhibitors promoted cell proliferation and Cyclin D1 expression of MKN28 cells (Fig. 4e, III and IV). These results suggested that the ERK-P65-miR23a/27a/24 axis played a suppressive role in GC progression.

### Low blood gastrin was correlated with poor prognosis of the GC patients and decreased expression of p-ERK and p-P65 in GC tissues

**Fig. 5:**
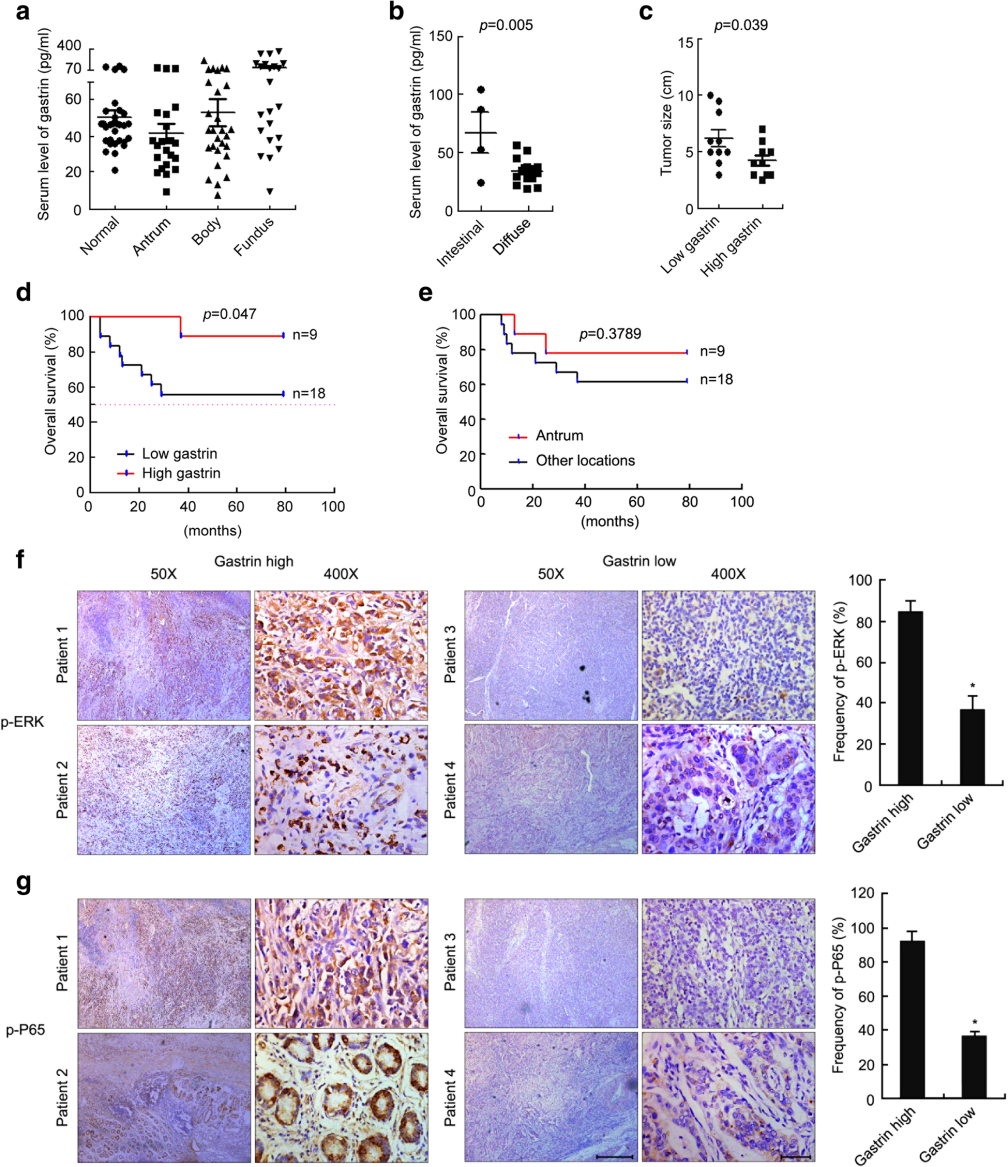
Low blood gastrin was correlated with GC poor prognosis and increased p-ERK and p-P65 expression. **a** Serum gastrin levels in GC patients, which were determined using ELISA. Normal, *n* = 30; Antrum, *n* = 22; Body, *n* = 28; Fundus, *n* = 22. **b** Serum gastrin levels of the patients with diffuse tumors (*n* = 15) and those arising from intestinal sinuses (*n* = 4). **c** Differential GC tumor sizes in GC patients with high (*n* = 10) and low (*n* = 10) serum gastrin levels. **d** Differential overall survival of GC patients with high (*n* = 9) and low (*n* = 18) serum gastrin levels, as revealed by Kaplan-Meier plots. *p* value was calculated by a log-rank test. **e** Differential overall survival of GC patients with antrum (n = 9) and other locations (*n* = 18), as revealed by Kaplan-Meier plots. *p* value was calculated by a log-rank test. **f**, **g** The association between serum gastrin levels and p-ERK and p-P65 expression in GC tissues. The levels of p-ERK (**f**) and p-P65 (**g**) in GC tissue samples were determined using IHC. The representative images of 4 patients were showed. Scale bar = 50 μm. p-ERK was expressed in 11 out of 13 patients with high serum gastrin, and in 12 out of 32 patients with low serum gastrin. p-P65 was expressed in 12 out of 13 patients with high serum gastrin, and in 12 out of 32 patients with low serum gastrin

To determine whether the ERK-P65-miR23a/27a/24 axis mediated suppression of PGC growth by gastrin, we first measured serum gastrin levels in GC patients using ELISA. The results showed that the serum gastrin levels were lower in patients with antrum GC and higher in patients with fundus GC than those of the healthy control groups (Fig. 5a). The serum gastrin levels were lower in the diffuse GC subtype than intestinal subtype (Fig. 5b). Larger tumors were found in the groups of GC patients with lower gastrin levels (Fig. 5c). GC patients with higher levels of gastrin had a longer overall survival (OS) time than those with lower gastrin levels (Fig. 5d). There was no significant difference in the overall survival between the patients with antrum GC and those with GC of other locations (Fig. 5e). These data suggested that low serum gastrin was correlated with poor prognosis of the GC patients. We further examined p-ERK and p-P65 levels in the paraffin-embedded tissues of GC patients by IHC. The results showed that higher frequencies of p-ERK and p-P65 expression were found in GC tissues of the patients with the higher serum gastrin levels (Fig. 5f and g).

### Gastrin inhibited proliferation of PGC cells through activating the ERK-P65-miR23a/27a/24 axis

**Fig. 6:**
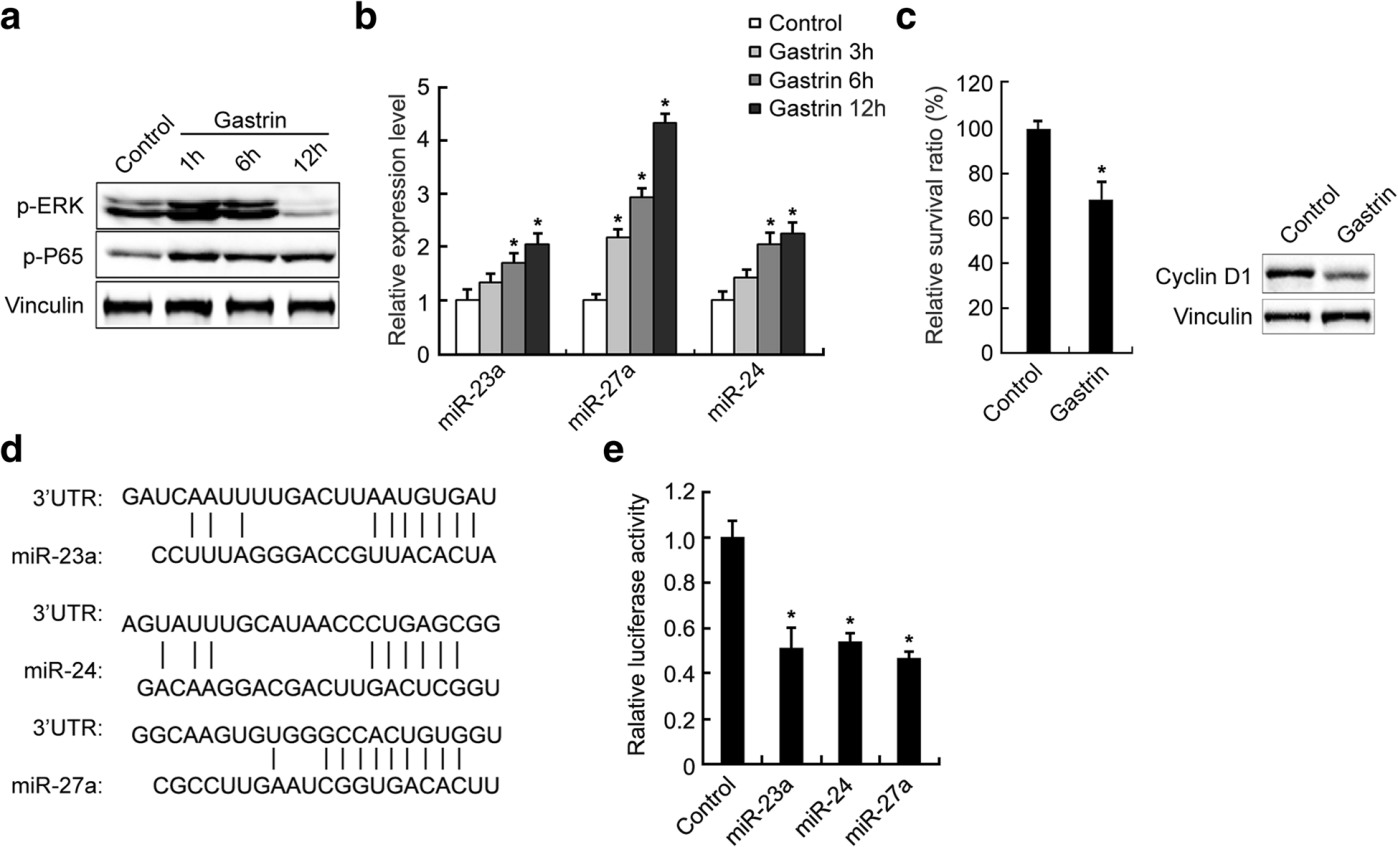
Gastrin inhibited GC cell proliferation through activating the ERK-P65-miR23a/27a/24 axis in vitro. The p-ERK, p-P65 (**a**), and miR-23a, miR-27a, and miR-24 (**b**) levels were increased and cyclin D1 levels and proliferation of SGC7901 cells (**c**) were decreased after gastrin treatment. SGC7901 cells were treated with 10^− 7^ mol/L gastrin for indicated duration. The p-ERK, p-P65, and cyclin D1 levels were determined using Western blotting. MicroRNA levels were determined by qRT-PCR and normalized to U6. The fold change was calculated using the 2^−ΔΔCt^ method. Proliferation of SGC7901 cells were determined by living cell counting. **d** Sequence alignment between cyclin D1 (CCND1) 3’ UTR and miR-23a, miR-27a, and miR-24 using Vector NTI 6.0 software. **e** The miR-23a, miR-24, and miR-27a mimics suppressed luciferase activity ofCCND1 3′-UTR reporter gene after cotransfectionin to HEK293T cells. *Renilla* luciferase reporter gene was used as normalization control for transfection efficiency. *, compared with the control group, *p*<0.05. All experiments were repeated three times

To further determine whether the ERK-P65-miR23a/27a/24 axis mediated suppression of PGC growth by gastrin, we next treated SGC7901 cells with gastrin and determined p-ERK and p-P65 levels using Western blotting, and miR-23a, miR-27a, and miR-24 levels using qRT-PCR. The results showed that p-ERK and p-P65 levels (Fig. 6a) and miR-23a, miR-27a, and miR-24 levels (Fig. 6b) were increased, and proliferation of SGC7901 cells and Cyclin D1 expression were inhibited (Fig. 6c) in SGC7901 cells after gastrin treatment. There was a good pairing between these three miRNAs and cyclin D1 3’ UTR (Fig. 6d). The miR-23a, miR-24 and miR-27a mimics suppressed luciferase activity ofCCND1 3′-UTR reporter gene after cotransfection into HEK293T cells (Fig. 6e). We also treated MKN45 cells with gastrin and determined p-ERK and p-P65 levels using Western blotting and found the consistent results with those of SGC7901 cells (Additional file 4: Figure S2A and B). These results suggested that inhibition of PGC cells proliferation by gastrin was probably mediated by activation of the ERK-P65-miR23a/27a/24 axis.

### Gastrin inhibited GC growth through activating the ERK-P65-miR23a/27a/24 axis in mice

**Fig. 7:**
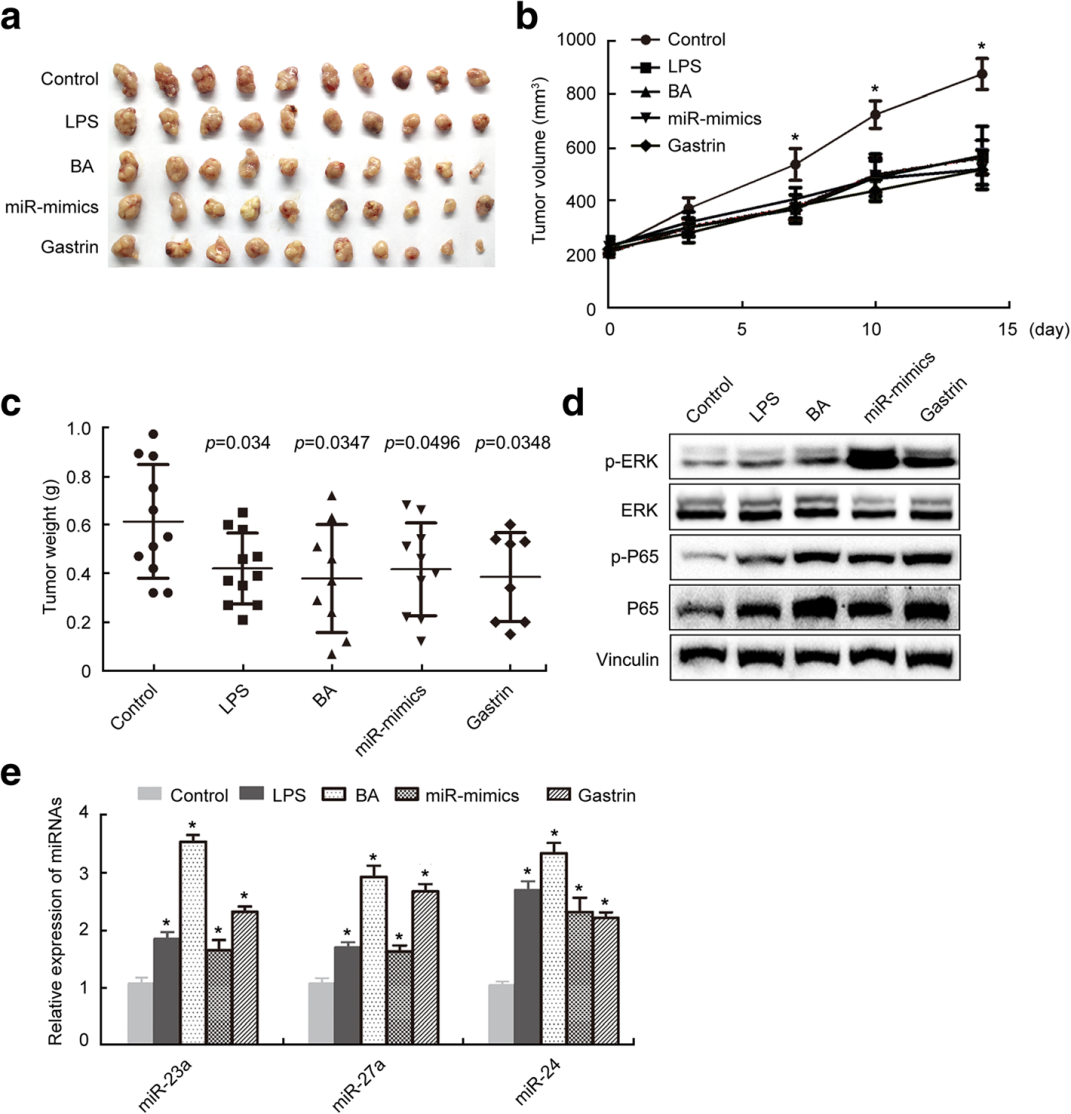
Gastrin and ERK-P65-miR23a/27a/24 axis activators inhibited growth of SGC7901 tumor tissues in mice. **a** The tumor tissues isolated from mice xenografted with SGC7901 cells (5 × 10^6^) and then treated with LPS, BA, miR23a/27a/24 mimics, and gastrin for 12 days. The volume (**b**) and weight (**c**) of tumor tissues were measured. Protein levels were determined using Western blotting (**d**). MicroRNA levels were determined using qRT-PCR with U6 as a normalization control. The fold change was calculated using the 2^−ΔΔCt^ method (**e**). *, compared with the control, *p*<0.05. The experiments were repeated three times

**Fig. 8:**
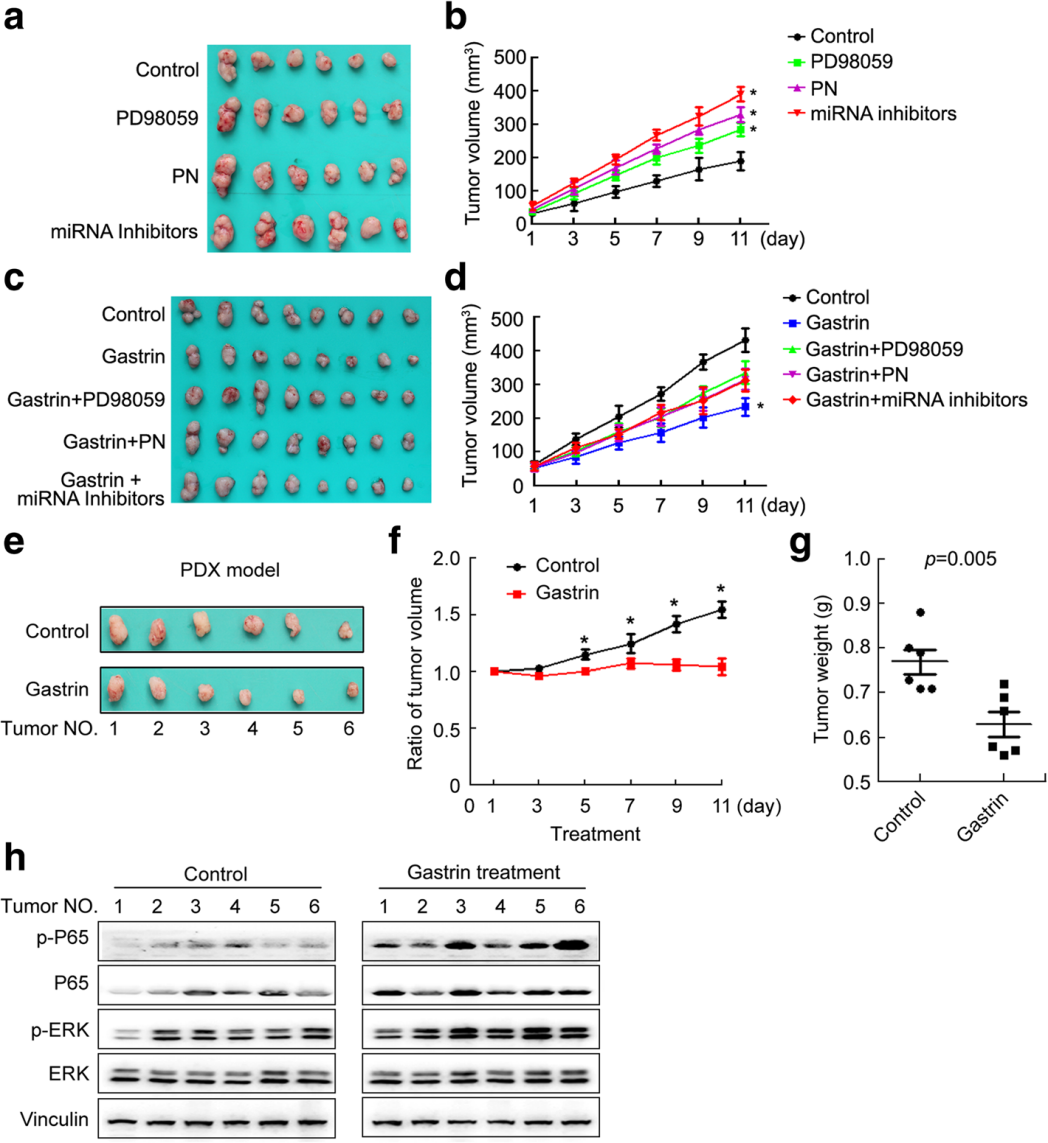
Gastrin inhibited tumor growth induced byERK-P65-miR23a/27a/24 axis inhibitors in a PDX mice model. **a**, **c** The tumor tissues isolated from mice xenografted with SGC7901 cells (5 × 10^6^) and then treated with PD98059, PN, and miR23a/27a/24 inhibitors (**a**) or combination with Gastrin for 11 days (**c**). The volume of tumor tissues was measured (**b**, **d**). **e**-**h** The tumor tissues isolated from PDX mice models treated either with gastrin (2 mg/kg, subcutaneous injection, twice a day) or 100 μl PBS/mouse/day (**e**). The volume (**f**) and weight (**g**) of the tumor tissues were measured. ERK, P65, p-ERK, and p-P65 protein levels were determined using Western blotting (**h**). *, compared with the control group, *p*<0.05. All measurements were repeated three times

To determine whether the ERK-P65-miR23a/27a/24 axis mediated suppression of PGC growth by gastrin in vivo, we treated a subcutaneous xenograft GC mouse model with gastrin, LPS, BA, and miRNA mimics, or with PD98059, PN, miR23a/27a/24 inhibitors, and their combination with gastrin, then examined the tumor volume and weight, ERK, p-ERK, P65, p-P65, miR-23a, miR-27a, and miR-24 levels in tumor tissues. The results showed that the tumor volume and weight were significantly inhibited (Fig. 7a-c), p-ERK, P65, p-P65, miR-23a, miR-27a, and miR-24 levels in tumor tissues were significantly increased(Fig. 7d and e) in mice after treatment with gastrin, LPS, BA, and miRNA mimics. The tumor volumes were significantly increased in mice after by PD98059, PN and miR23a/27a/24 inhibitors treatment (Fig. 8a and b). Co-treatment of mice with gastrin and PD98059, PN, or miR23a/27a/24 inhibitors resulted significant decreases in the tumor volumes, compared with the vehicle control groups (Fig. 8c and d). We also examined the effects of gastrin on the PGC PDX model. The results showed that the tumor volume and weight were significantly decreased (Fig. 8e-g) and the tumor tissue p-ERK, P65, and p-P65 levels were increased (Fig. 8h) in the PGC PDX mice after gastrin treatment, compared with the vehicle control group. These data supported that gastrin inhibited GC growth through activating the ERK-P65-miR23a/27a/24 axis in mice.

### Gastrin enhanced the suppression of GC by cisplatin in mice model

**Fig. 9:**
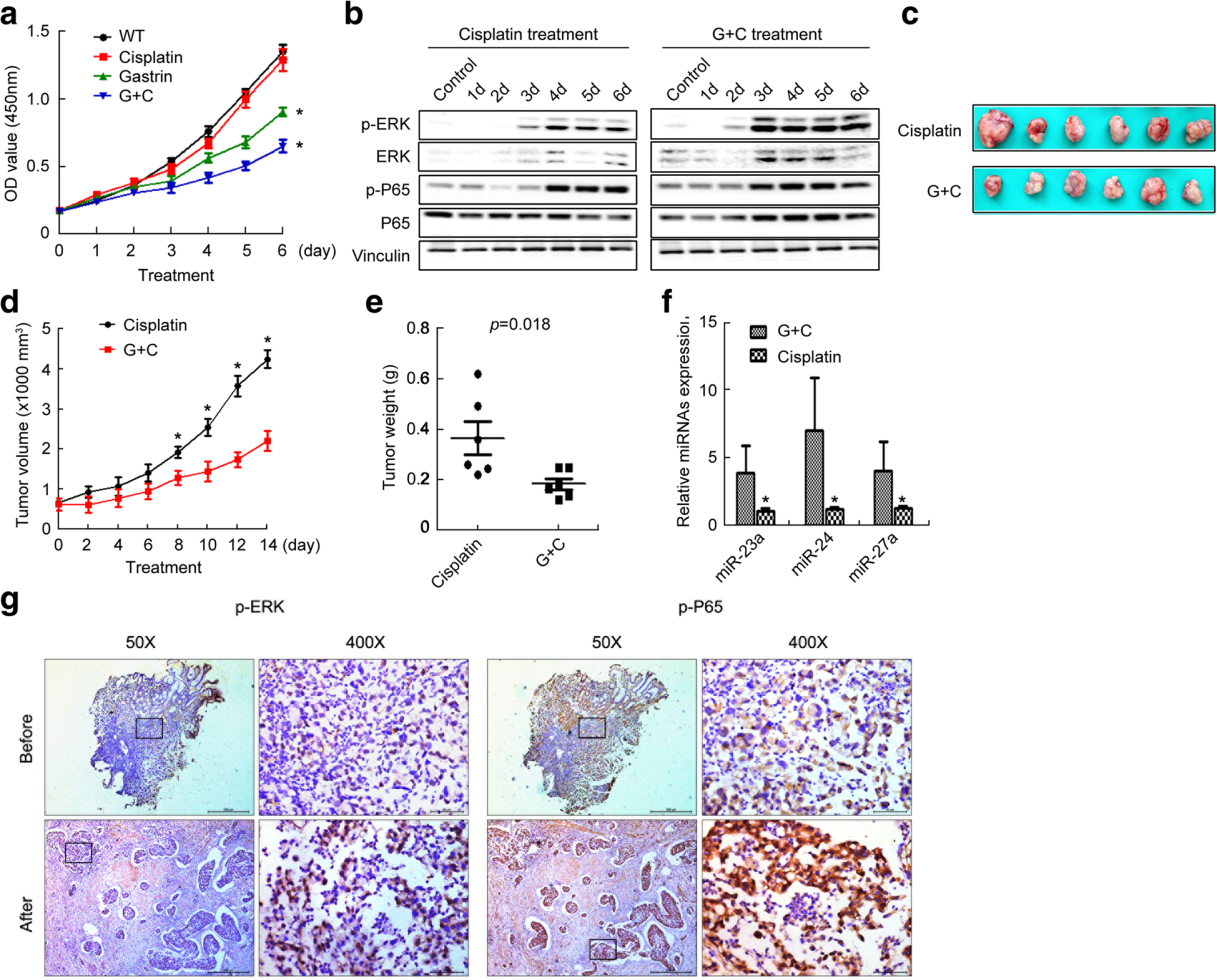
Gastrin enhanced the inhibitory effect of cisplatin GC growth. **a**-**b** GC cells were treated with gastrin (1 nM), cisplatin (2 mM) or combination. The media containing indicated drugs were replaced every day. The cell proliferation was determined by a CCK-8 assay (**a**), and the protein levels were determined using Western blotting (**b**). **c**-**f** The tumor tissues isolated from mice xenografted with SGC7901 cells (5 × 10^6^) and then treated with PD98059, PN, and miR23a/27a/24 inhibitors for 11 days (Gastrin and cisplatin vs cisplatin,14 day) (**c**). The volume (**d**) and weight (**e**) of the tumor tissues were measured. The miR-23a, miR-27a and miR-24 levels were determined by qRT-PCR. **f** *, compared with the control group, *p*<0.05. **g** Changes of p-ERK and p-P65 levels in GC tissues of patients after treatment with Oxaliplatin and Tegafur**.** The p-ERK and p-P65 levels were determined by IHC. The representative images of 4 patients were showed. Scale bar = 50 μm

To determine whether gastrin enhances the suppression of GC by cisplatin, we first treated SGC7901 cells with gastrin, cisplatin, and the combination, and examined proliferation of SGC7901 cells. The results showed that treatment with both gastrin and cisplatin resulted in more significant suppression of SGC7901 cells proliferation, compared with that of treatment with cisplatin, gastrin, or vehicle alone (Fig. 9a). It also resulted in more significant increases in the levels of ERK, p-ERK, P65, and p-P65, compared with that of treatment with cisplatin (Fig. 9b). We also treated the PGC PDX mice with cisplatin and the combination of gastrin and cisplatin, and examined the tumor volume and weight as well as miR-23a, miR-27a and miR-24 expression in the tumor tissues. The results showed that treatment with both gastrin and cisplatin resulted in decreases in the tumor size and tumor weight (Fig. 9c-e), increases in miR-23a, miR-27a and miR-24 levels (Fig. 9f), compared with treatment with cisplatin alone. In addition, effective neoadjuvant chemotherapy with Oxaliplatin and Tegafur clinically led to the up-regulation of p-ERK and p-P65 in GC tissues (Fig. 9g). These results suggested that gastrin enhanced the suppression of GC by cisplatin in mice model probably through the ERK-P65-miR23a/27a/24 axis.

## Discussion

In the current study, we have confirmed the presence of ERK- P65-miR23a/27a/24 axis in GC cells and found that the levels of components of ERK-P65-miR23a/27a/24 axis are decreased in GC tissue samples and PGC cells. The decreased levels of components of ERK-P65-miR23a/27a/24 axis are associated with poor prognosis of GC, and ERK-P65-miR23a/27a/24 axis plays a suppressive role in GC progression. These data support that ERK-P65-miR23a/27a/24 axis is down-regulated, leading to excess GC growth and poor prognosis of GC. Further, we showed that low blood gastrin was correlated with poor prognosis of the GC patients and decreased expression of p-ERK and p-P65 in GC tissues. Gastrin inhibited proliferation of PGC cells through activating the ERK-P65-miR23a/27a/24 axis. Gastrin inhibited GC growth and enhanced the suppression of GC by cisplatin in mice or PGC cell culture models through activating the ERK-P65-miR23a/27a/24 axis or its components. These data suggested that low gastrin promoted excess GC growth and contributed to the poor prognosis of the GC patients by down-regulating ERK-P65-miR23a/27a/24 axis.

In a recent study, we found that P65 upregulated miR23a/27a/24 expression. Sustained activation of P65 and up-regulation of miR23a/27a/24expression silenced the expression of anion exchanger 1 (AE1), a major membrane protein in RBC. The absence of AE1 is associated with differentiation arrest of erythroleukemia K562 cells. This pathogenesis provided a molecular basis for leukemia that allowed the development of protein kinase inhibitors such as imatinib and dasatinib that are therapeutically effective [27]. In the current study, we confirmed the presence of ERK-P65-miR23a/27a/24 axis in GC cells. The decreased levels of components of ERK-P65-miR23a/27a/24 axis are associated with poor prognosis of GC, and ERK-P65-miR23a/27a/24 axis plays a suppressive role in GC progression. It has been shown that AE1 was expressed in GC cells and is associated with GC pathogenesis by promoting sequestration of P16 in the cytoplasm and promoting alkalization of GC cells [44].

In the current study, we showed that low blood gastrin was correlated with poor prognosis of the GC patients. Gastrin inhibited GC growth and enhanced the suppression of GC by cisplatin in mice or PGC cell culture models. This is consistent with our previous study that gastrin inhibits PGC growth in vitro and in vivo [30]. Several studies have shown that gastrin promotes tumor growth based on clinical observation or animal models of hypergastrinemia [31–34]. This is different from our findings in the current study. Therefore, the effects of gastrin on GC progression remains controversial [35–37], probably due to inconsistent factors such as specimens, ethnics, genetic background, and environmental factors. Gastric cancer is a heterogeneous disease with a variety of predisposing and etiologic factors, and there is difference between “Eastern” and “Western” gastric cancer, including histology, tumor location, baseline patient characteristics, environmental and dietary factors, and Helicobacter pylori status. A much higher incidence of tumors located in the proximal third of the stomach was found in the Western, while much preferred to located in distal stomach in the Eastern where the production and secretion of gastrin were destroyed, leading to the lower serum gastrin level in Asian. The prevalence of diffuse histology is higher in the Western GC patients. There was a significant association of higher serum gastrin level and intestinal metaplasia [45]. Our results showed that serum gastrin level was lower in diffuse type GC. The environmental factors and dietary habits between the Eastern and Western people are quite different, resulting differences in the prevalence of obesity, diabetes, and tobacco use, which are associated with increased perioperative complications in gastric cancer as well as many other tumors [46, 47]. The profiling of cancer mutation genes has identified significant differences in APC, ARIDIA, KMT2A, PIK3CA, and PTEN genes between GC patients of Asian and Caucasian [48]. The in-depth mechanistic investigations on gastric cancer biology and/or host factors are necessary to fully understand the difference between the Western GC and the Eastern GC.

Since gastrin was dominantly synthesized in the G cells of the antrum, the secretion of gastrin was destroyed in patients with antrum GC, resulting in decreased gastrin levels, as we observed that low gastrin levels in patients with antrum GC and up-regulated gastin levels in patients with other sites (body and fundus) of GC due to the compensatory effects (Fig. 5a and b). This was consistent with the other published results [45]. We then investigated and found that low blood gastrin was correlated with tumor sizes (Fig. 5c) and poor prognosis (Fig. 5d) of the GC patients. These data suggested that the gastrin levels were associated with the locations or types of GC and correlated with the prognosis (Fig. 5a-d). It is highly reasonable to speculate that locations of GC could be associated with the prognosis of GC. To test this hypothesis, we analyzed differential overall survival of GC patients with antrum and other locations using Kaplan-Meier plots, and found that there was no significant difference in the overall survival between the patients with antrum GC and those with GC of other locations (Fig. 5e). Whether the location of GC is correlated with the prognosis of GC deserves further investigation by including more GC patients in the future study.

To investigate the underlying mechanism for gastrin to inhibit GC progression found in this study, we showed that ERK-P65-miR23a/27a/24 axis was down-regulated in GC, leading to excess GC growth and poor prognosis of GC and that gastrin inhibited GC growth and enhanced the suppression of GC by cisplatin in mice or PGC cell culture models in the current study. Consistently, we found that low blood gastrin was correlated with poor prognosis of the GC patients and decreased expression of p-ERK and p-P65 in GC tissues. Gastrin activated the ERK-P65-miR23a/27a/24 axis and inhibited proliferation of PGC cells. Further, gastrin activated the ERK-P65-miR23a/27a/24 axis or its components and inhibited GC growth and enhanced the suppression of GC by cisplatin in mice or PGC cell culture models. This observation is consistent with the previous studies showing that miR-27a suppresses ZBTB10/RINZF genes expression [49] and ZBTB10/RINZF inhibits the Sp1-depedent transcription of gastrin [50]. It seems that gastrin and miR-27a are mutually up-regulated. These data support that ERK-P65-miR23a/27a/24 axis mediates the suppressive effects of gastrin on GC growth, providing a mechanism for gastrin to inhibit GC progression. How gastrin activates the ERK-P65-miR23a/27a/24 axis or its components remains to be investigated in the future.

Cisplatin is a first-line chemotherapeutic agent for GC treatment [51–54]. However, it is very toxic and confer great adverse side effects when applied to GC treatment [55, 56]. In the current study, we found that gastrin enhanced the suppressive effects of cisplatin on GC in comparison with treatment with gastrin or cisplatin alone. Therefore, a gastrin-cisplatin combination therapy would promote efficacy and reduce toxicity of cisplatin therapy of GC by allowing lower cisplatin doses to be used.

It has been shown that several signaling pathways are involved in GC progression, including MAPK (ERK, JNK, P38) [57–59], PI3K-Akt-mTOR [60–64], AMPK-mTOR [65, 66], COX-2/NF-κB [67–70], Wnt signaling pathways [71–74]. Non-coding RNAs including miRNA and lncRNA play important roles in gastric cancer progression or drug resistance. They may also serve as biomarker for diagnosis or targeted therapy of GC, such as miR-21 [75–82]. For the first time, we found that ERK-P65-miR23a/27a/24 axis is involved in GC progression, providing an additional mechanism or target to investigate novel approaches for GC therapy.

In the current study, we classified the GC patients into intestine, diffuse, and mixed type using the classic Lauren method and investigated the role of gastrin in GC growth and development. The Cancer Genome Atlas (TCGA) project has mapped a genomic landscape of GC and described four groups of gastric cancer based upon molecular classifications, including the EBV (Epstein-Barr virus), MSI (microsatellite instability), GS (genomically stable), and CIN (chromosomal instability) types [83]. It is very intriguing to investigate the association and the underlying molecular mechanism of gastrin with the TCGA GC types in our future studies.

## Conclusions

Collectively, we have found that ERK-P65-miR23a/27a/24 axis is down-regulated in GC, which may play a role in excess GC growth and poor prognosis of GC. Low gastrin promotes excess GC growth and contributes to the poor prognosis of the GC patients by down-regulating ERK-P65-miR23a/27a/24 axis. Gastrin inhibits GC progression and activates ERK-P65-miR23a/27a/24 axis which functions as a GC suppressor. Gastrin and the ERK-P65-miR23a/27a/24 axis could be a potential drug target for PGC therapy.

## Additional files

Additional file 1: **Table S1.** Association of ERK expression and clinicopathological features of GC. (DOC 44 kb)

Additional file 2: **Table S2.** Association of P65 expression and clinicopathological features of GC. (DOC 49 kb)

Additional file 3: **Figure S1.** LY3214996 promoted the proliferation of MKN45 cells. (TIF 44 kb)

Additional file 4: **Figure S2.** Gastrin inhibited the proliferation of MKN45 cells. (TIF 66 kb)

## Abbreviations

AE1: Anion exchanger 1
BA: Betulinic acid
BRAF: v-Raf murine sarcoma viral oncogene homolog B
CAG: Chronic atrophic gastritis
CCK-8: Cell Counting Kit-8
CCK-B: Cholecystokinin-B
CIN: Chromosomal instability
DAB: Diaminobenizidine
DIG: Digoxidenin
EBV: Epstein-Barr virus
ELISA: Enzyme linked immunosorbent assay
ERK: Extracellular signal-regulated kinase
FBS: Fetal bovine serum
GC: Gastric cancer
GS: Genomically stable
HSC: Hemopoietic stem cells
IHC: Immunohistochemistry
LNA: Locked nucleic acid
LPS: Lipopolysaccharides
MAPK: Mitogen-activated protein kinase
MEK: Mitogen-activated protein kinase kinase
MSI: Microsatellite instability
NBT-BCIP: Nitroblue tetrazolium/5-Bromo-4-Chloro-3-Indolyl Phosphate
OS: Overall survival
PBS: Phosphate buffer saline
PDX: Patient-derived xenografts
PGC: Poorly-differentiated GC
PN: Parthenolide
qRT-PCR: Quantitative real time PCR
RAS: Rat sarcoma virus
TBST: Tris-buffered Saline with Tween 20
TCGA: The Cancer Genome Atlas
TMA: Tissue microarray
TNM: Tumor, node, metastasis
WGC: Well-differentiated GC
