# Supplementary material for: Gastrin inhibits gastric cancer progression through activating the ERK-P65-miR23a/27a/24 axis
Source: J Exp Clin Cancer Res. 2018 Jun 4;37:115. doi: 10.1186/s13046-018-0782-7 (PMC5987590; doi:10.1186/s13046-018-0782-7)
Supplement: Supplementary file 1 — Table S1. Association of ERK expression and clinicopathological features of GC. (DOC 44 kb) [file 13046_2018_782_MOESM1_ESM.doc]

| Clinicopathological features | | N | ERK protein expression | | *p* value |
| --- | --- | --- | --- | --- | --- |
|  |  |  | Negative | * * Positive |  |
| Gender | Male | 61 | 9（14.75%） | 52（85.25%） | 0.275 |
|  | Female | 37 | 2（5.41%） | 35（94.59%） |  |
| Age (years) | ≦57 | 42 | 12（28.57%） | 30（71.43%） | 0.929 |
|  | ＞57 | 51 | 15（29.41%） | 36（70.59%） |  |
| Tumor diameter | ≦4cm | 26 | 5(19.23%) | 21(80.77%) | **0.017** |
|  | ﹥4cm | 58 | 27（46.55%） | 31（53.45%） |  |
| Lymphatic metastasis | (-) | 32 | 12(37.50%) | 20(62.50%) | 0.188 |
|  | (+) | 65 | 16(24.62%) | 49(75.38%) |  |
| Differentiation | Well | 35 | 7（20.00%） | 28（80.00%） | **0.008** |
|  | Poorly | 66 | 31（46.97%） | 35（53.03%） |  |
| Lauren classification | Intestinal type | 48 | 5（10.42%） | 43（89.58%） | **0.000** |
|  | Diffuse type | 57 | 24（42.12%） | 29（50.88%） |  |

Additional file 1: Table S1. Association of ERK expression and clinicopathological features of GC. ERK expression was detected in TMA using IHC.

Values in parenthesis are percentage;

Bold fonts represent the value was statistically significant;
