# Supplementary material for: Gastrin inhibits gastric cancer progression through activating the ERK-P65-miR23a/27a/24 axis
Source: J Exp Clin Cancer Res. 2018 Jun 4;37:115. doi: 10.1186/s13046-018-0782-7 (PMC5987590; doi:10.1186/s13046-018-0782-7)
Supplement: Supplementary file 2 — Table S2. Association of P65 expression and clinicopathological features of GC. (DOC 49 kb) [file 13046_2018_782_MOESM2_ESM.doc]

| Clinicopathological features | | No. | P65 protein expression | | *p* value |
| --- | --- | --- | --- | --- | --- |
|  |  |  | Negative No. (%) | * * Positive No. (%) |  |
| Gender | Male | 50 | 18(36.00%) | 32(64.00%) | 0.798 |
|  | Female | 36 | 12(33.33%) | 24(66.67%) |  |
| Age (years) | ≦57 | 33 | 15(45.46%) | 18(54.54%) | 0.105 |
|  | ＞57 | 53 | 15(28.30%) | 38(71.70%) |  |
| Tumor diameter | ＜4cm | 34 | 9(26.47%) | 25(73.52%) | **0.025** |
|  | ≧4cm | 36 | 19(52.78%) | 17(47.22%) |  |
| Invasion depth | T1~T3 | 24 | 10(41.67%) | 14(58.33%) | 0.202 |
|  | T4 | 62 | 17(27.42%) | 45(72.58%) |  |
| Lymphatic metastasis | (-) | 26 | 7(26.92%) | 19(73.08%) | 0.463 |
|  | (+) | 60 | 21(35.00%) | 39(65.00%) |  |
| TNM stage | I-II | 45 | 18(40%) | 27(60%%) | 0.916 |
|  | III-IV | 34 | 14(41.18%) | 20(58.82%) |  |
| Differentiation | Well | 31 | 7(22.58%) | 24(77.42%) | **0.023** |
|  | Poorly | 57 | 27(47.34%) | 30(52.63%) |  |
| Lauren classification | Intestinal type | 40 | 10(25.00%) | 30(75.00%) | **0.029** |
|  | Diffuse type | 46 | 22(47.83%) | 24(52.17%) |  |

Additional file 2: Table S2. Association of P65 expression and clinicopathological features of GC. P65 expression was detected in TMA using IHC.

Values in parenthesis are percentage;

Bold fonts represent the value was statistically significant;
