# Supplementary figures and images for: Gastrin inhibits gastric cancer progression through activating the ERK-P65-miR23a/27a/24 axis
Source: J Exp Clin Cancer Res. 2018 Jun 4;37:115. doi: 10.1186/s13046-018-0782-7 (PMC5987590; doi:10.1186/s13046-018-0782-7)

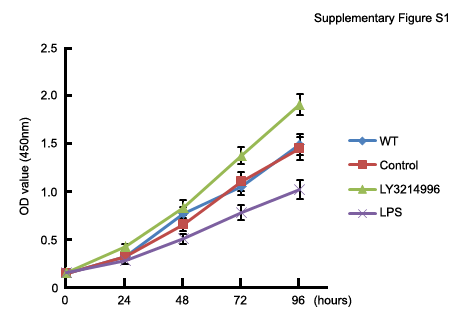

Supplement: Supplementary file 3 — Figure S1. LY3214996 promoted the proliferation of MKN45 cells. (TIF 44 kb) [file 13046_2018_782_MOESM3_ESM.tif]

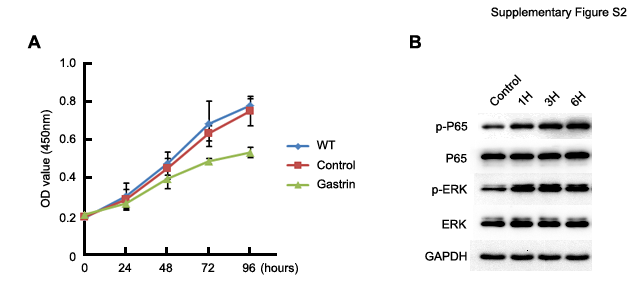

Supplement: Supplementary file 4 — Figure S2. Gastrin inhibited the proliferation of MKN45 cells. (TIF 66 kb) [file 13046_2018_782_MOESM4_ESM.tif]
